# Developmental Stage Shapes the Leaf-Associated Bacterial Community of *Eugenia uniflora* and Reveals Shared Bacterial Taxa with Other Myrtaceae Species

**DOI:** 10.3390/plants15203138

**Published:** 2026-10-14

**Authors:** Isabel Cristina Cadavid Sánchez, Dámaris Esquén Bayona, Belkys Brianna Medrano Salazar, Rogerio Margis, Frank Guzman Escudero

**Affiliations:** 1 Departamento de Biofisica, Centro de Biotecnologia, Universidade Federal do Rio Grande do Sul, Porto Alegre 91501-970, Brazil; isacadavid9@gmail.com (I.C.C.S.); rogerio.margis@ufrgs.br (R.M.); 2 Departamento de Genética, Universidade Federal do Rio Grande do Sul, Porto Alegre 91501-970, Brazil; 3 Laboratorio de Biomoléculas, Facultad de Ciencias de la Salud, Universidad Peruana de Ciencias Aplicadas, Lima 15067, Peru; esquendamaris@gmail.com (D.E.B.); belkysmedrano7@gmail.com (B.B.M.S.); 4 Escuela de Biología, Facultad de Ciencias de la Salud, Universidad Peruana de Ciencias Aplicadas, Lima 15067, Peru

**Keywords:** endophytes, epiphytes, amplicon sequence variants (ASVs), microbial diversity, plant–microbe interactions, functional prediction potential, network analysis, Atlantic Forest, 16S rRNA gene sequencing

## Abstract

The phyllosphere microbiome contributes to plant health and adaptation, yet bacterial communities associated with *Eugenia uniflora* remain poorly characterized. This study investigated how developmental stage shapes the *E. uniflora* phyllosphere microbiome and assessed bacterial taxa and associations shared with other Myrtaceae species. The V3–V4 region of the 16S rRNA gene was sequenced from 19 *E. uniflora* samples representing young and mature trees, as well as 13 samples from mature trees representing three additional Myrtaceae genera (*Myrcianthes*, *Plinia*, and *Psidium*) and other *Eugenia* species. Community diversity, predicted functional potential, co-occurrence networks, and core taxa were analyzed. In *E. uniflora*, 1428 bacterial ASVs belonging to 17 phyla and 135 genera were identified, and bacterial community composition differed significantly between developmental stages. Young and mature trees showed distinct taxonomic profiles and predicted functional patterns. Network analysis revealed more connected bacterial communities in young plants and simpler networks in mature plants. Comparison with other Myrtaceae identified 17 core genera, including *Methylobacterium–Methylorubrum*, *Hymenobacter*, *Sphingomonas*, *Bdellovibrio*, and *Terriglobus*, which occurred in all 21 mature trees. Overall, developmental stage was associated with changes in the taxonomic, predicted functional potential, and network organization of the *E. uniflora* phyllosphere microbiome, while shared bacterial taxa and associations across the sampled Myrtaceae genera suggest conserved features of phyllosphere community assembly.

## 1. Introduction

The plant microbiome encompasses the community of microorganisms that colonize internal and external plant compartments, playing vital roles in plant health and development [1,2]. These microbial communities support nutrient acquisition through processes such as nitrogen fixation and mineral solubilization, promote plant growth through phytohormone production, and enhance tolerance to biotic and abiotic stresses by activating plant defense mechanisms and suppressing pathogens [3,4,5]. Owing to these beneficial functions, plant-associated microorganisms are increasingly being explored for the development of bioinoculants and other sustainable agricultural technologies [6].

The phyllosphere, which comprises all aboveground plant surfaces (including leaves, stems, flowers, and fruits), is one of the largest microbial habitats on Earth. As the primary interface between plants and the atmosphere, it harbors diverse communities of bacteria and fungi, that constitute an important component of the plant’s first line of defense against environmental stress and pathogen invasion [7,8]. Beneficial microorganisms inhabiting the phyllosphere play essential roles in plant health and ecosystem functioning by increasing tolerance to heat, desiccation, and ultraviolet radiation, suppressing pathogens through resource competition and antimicrobial compound production, and promoting plant growth through phytohormone synthesis. In addition, these microbial communities contribute to global biogeochemical cycles by participating in nitrogen fixation, degradation of volatile organic compounds, and plant biomass decomposition [9,10]. The phyllosphere microbiome comprises microorganisms associated with the leaf surface (epiphytes) and those residing within plant tissues (endophytes). Although these compartments may differ ecologically, both contribute to the overall leaf-associated microbial community. In this study, the leaf-associated bacterial community was characterized as a whole, without experimentally separating epiphytic and endophytic fractions.

The composition and diversity of plant-associated microbial communities are influenced by multiple factors, including host species, genotype, tissue type, developmental stage, and environmental conditions, and may reflect long-term ecological associations between plants and their associated microorganisms [11,12,13]. Changes in plant diversity resulting from domestication or environmental disturbances can alter these microbial communities [13,14], making the plant microbiome an important indicator of ecosystem health and a valuable source of microorganisms with biotechnological potential.

Among the factors shaping plant microbiomes, developmental stage is increasingly recognized as a major driver of microbial community assembly. As plants develop, they undergo dynamic physiological, metabolic, and immunological changes that alter the production of exudates and secondary metabolites, thereby influencing the recruitment and selection of specific microbial communities according to changing nutritional and defensive requirements [15,16,17]. Consequently, microbial community composition often changes throughout plant development, reflecting shifts in host physiology and ecological interactions.

Another important aspect of plant microbiome research is the identification of microbial taxa consistently associated with a particular host or group of hosts, commonly referred to as the core microbiome. Core microbiome members are typically defined based on their consistent occurrence across a predefined set of samples or hosts [18], and their identification can provide insights into patterns of microbiome assembly and host–microbe associations. Although consistent occurrence does not necessarily imply a stable or functional association, core taxa may include microorganisms that contribute to important processes such as nutrient acquisition, stress tolerance, plant defense, and pathogen suppression. Identifying bacterial taxa shared among related hosts can therefore help reveal conserved components of plant-associated microbiomes and identify microorganisms that warrant further investigation for their potential applications in sustainable agriculture [19].

Within this context, species of the Myrtaceae family are of particular ecological and economic importance. Native primarily to the Neotropics and Australia, Myrtaceae are abundant in biodiversity hotspots such as the Brazilian Atlantic Forest, where they provide essential food resources for insects, birds, and mammals through their flowers and fruits [20]. Numerous species also produce edible fruits and accumulate bioactive secondary metabolites, including flavonoids and terpenoids, that have recognized pharmacological properties [21,22,23].

Recent advances in high-throughput sequencing have begun to reveal the microbial communities associated with several Myrtaceae species. However, these studies remain limited to a small number of hosts [23,24,25], and comparative analyses across genera are still scarce. In particular, little is known about how plant developmental stage influences phyllosphere bacterial communities in native Myrtaceae species or whether closely related genera harbor conserved bacterial taxa despite differences in host identity. Consequently, the factors shaping phyllosphere microbiome assembly across Myrtaceae hosts remain incompletely understood.

*Eugenia uniflora* (commonly known as pitanga), a Myrtaceae species native to the Brazilian Atlantic Forest, exemplifies both the ecological and pharmacological importance of this family. Besides producing edible fruits, it is widely recognized for its medicinal properties and its production of biologically active secondary metabolites [26,27]. Despite its importance, the phyllosphere microbiome of *E. uniflora* remains poorly characterized, particularly using culture-independent, high-throughput sequencing approaches [23,24,25]. Moreover, the influence of host developmental stage on its phyllosphere bacterial community has not been investigated. To place the bacterial community of *E. uniflora* in a broader Myrtaceae context, mature *E. uniflora* trees were additionally compared with mature representatives of the genera *Myrcianthes*, *Plinia*, and *Psidium*, as well as other *Eugenia* species available at the sampling sites. These genera are represented among the diverse Myrtaceae documented in Brazilian flora [6,28]. The additional Myrtaceae taxa included in this comparison were primarily determined by their availability at the sampling sites. Because the available individuals of these additional hosts were mature, only mature *E. uniflora* trees were included in this comparative analysis to minimize potential differences in bacterial community composition associated with host developmental stage.

Therefore, the objectives of this study were to (i) characterize the total leaf-associated bacterial community of young (2-year-old) and mature (approximately 15- to 25-year-old) *E. uniflora* trees, encompassing both epiphytic and endophytic microorganisms; (ii) determine how developmental stage influences bacterial diversity, community composition, microbial interactions and predicted functional profiles, and (iii) identify bacterial taxa consistently shared among mature *E. uniflora* and eight additional mature Myrtaceae species. It was hypothesized that plant developmental stage significantly influences phyllosphere bacterial community assembly through age-dependent physiological changes and that phylogenetically related Myrtaceae genera share a subset of conserved bacterial taxa despite differences in their overall microbiome composition. By integrating developmental and comparative analyses, this study provides new insights into microbiome assembly in native Myrtaceae species and identifies bacterial taxa that represent promising candidates for future investigations on plant growth promotion and biological control.

## 2. Results

### 2.1. Bacterial Community Structure and Functional Potential Vary with Developmental Stage in Eugenia uniflora

**Figure 1:**
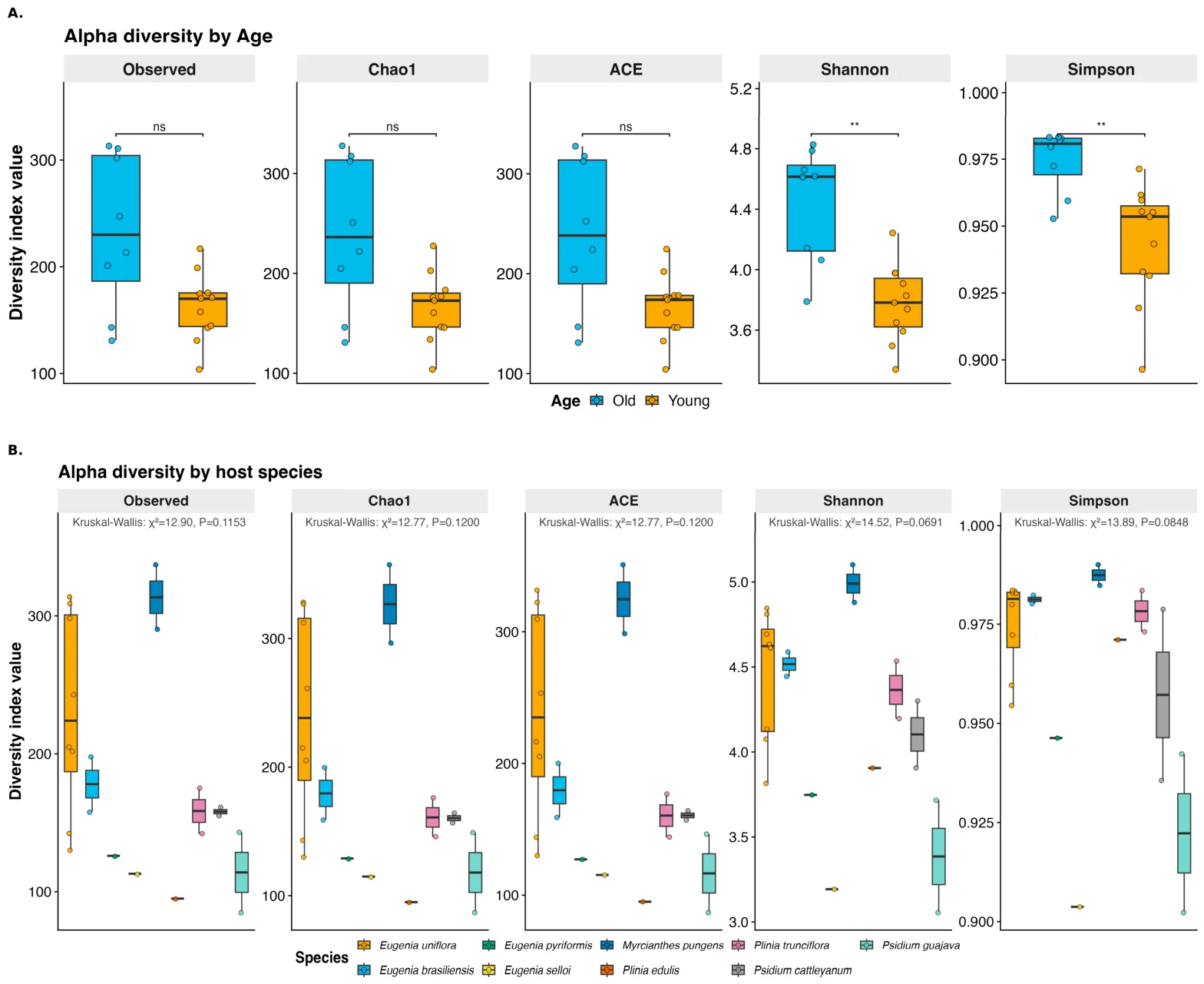
Alpha diversity of phyllosphere bacterial communities based on the Observed ASVs, Chao1, ACE, Shannon, and Simpson diversity indices. (**A**) Comparison between young and mature *Eugenia uniflora* trees. ns = not significant, ** *p* < 0.01; (**B**) Comparison among mature trees of the nine Myrtaceae species (*n* = 21). Each point represents an individual sample. Differences between young and mature trees (**A**) were evaluated using Wilcoxon rank-sum tests with Benjamini–Hochberg adjustment, and differences among host species (**B**) using the Kruskal–Wallis test; *p* < 0.05 was considered statistically significant.

The bacterial community associated with *Eugenia uniflora* comprised 1428 ASVs (Amplicon Sequence Variants, Table S1), which were used to assess microbial diversity. Shannon and Simpson diversity were significantly higher in mature than in young trees (Wilcoxon rank-sum test, Benjamini–Hochberg-adjusted *p* = 0.006 for both), whereas richness estimates did not differ significantly after adjustment (Observed ASVs, Chao1, and ACE; adjusted *p* = 0.052) (Figure 1A; Table S3). Richness was similar between mature and young trees within Morungava (median Observed ASVs, 166 vs. 170) and was higher in trees from Porto Alegre (median, 274.5), indicating that differences in richness were associated with sampling locality rather than with developmental stage.

**Figure 2:**
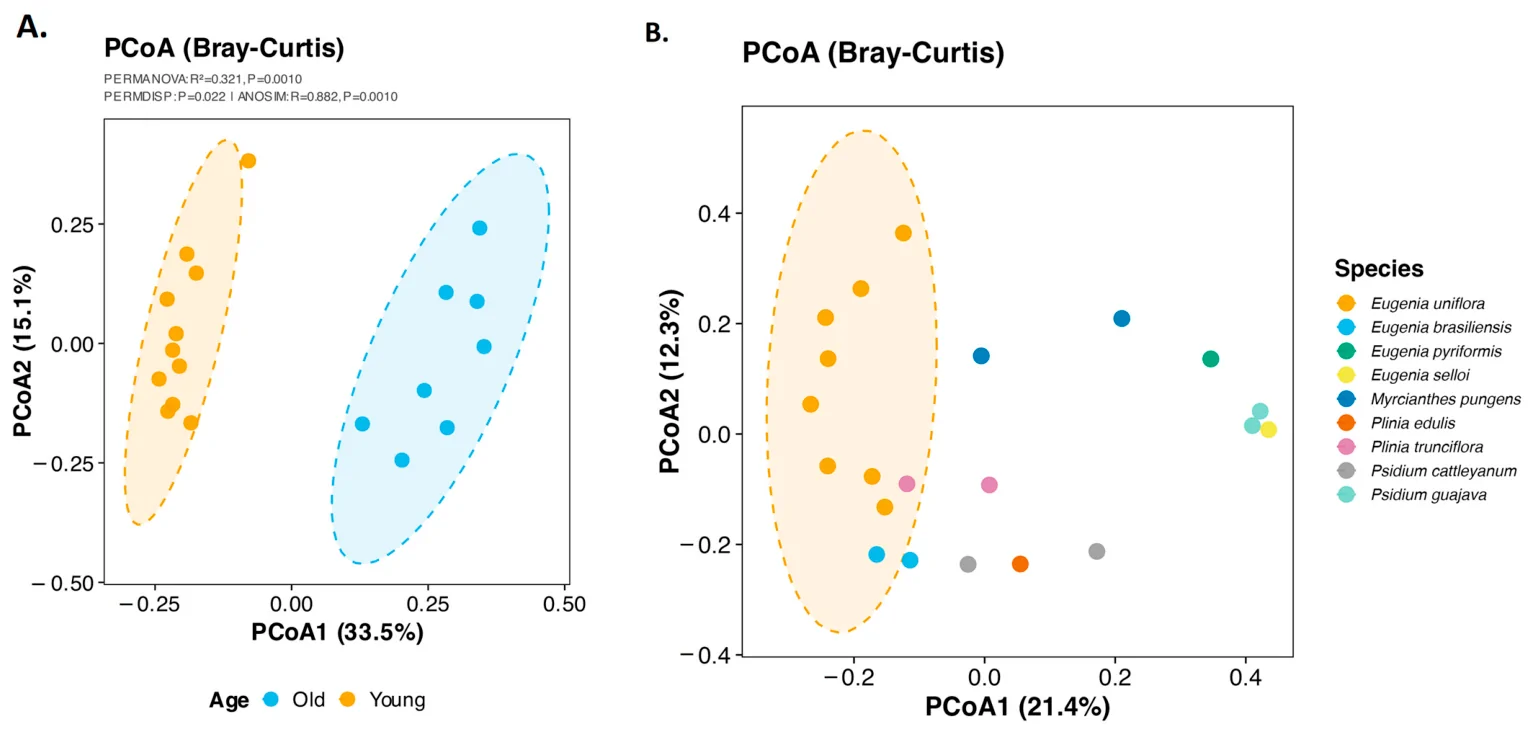
Principal Coordinates Analysis (PCoA) based on Bray–Curtis dissimilarity of phyllosphere bacterial communities. (**A**) *Eugenia uniflora* samples grouped according to developmental stage (mature and young trees). Differences in community composition were assessed using PERMANOVA based on Bray–Curtis distances (vegan::adonis2; F = 8.035, R^2^ = 0.321, *p* = 0.001, 999 permutations; PERMDISP, *p* = 0.022; ANOSIM, R = 0.882, *p* = 0.001). (**B**) Mature trees of the nine Myrtaceae species included in this study (*n* = 21). Differences in community composition were assessed by plant species using PERMANOVA based on Bray–Curtis distances (vegan::adonis2; F = 2.31, R^2^ = 0.607, *p* = 0.001, 999 permutations). Each point represents the bacterial community of an individual sample, and colors indicate developmental stage (**A**) or plant species (**B**). Dashed lines indicate 90% normal data ellipses (drawn only for groups with at least three samples).

Beta-diversity analysis revealed differences in bacterial community composition according to developmental stage (Figure 2A). Using Bray–Curtis dissimilarities and a PERMANOVA with developmental stage as the explanatory factor, developmental stage significantly explained 32.1% of the variation in bacterial community composition when all *E. uniflora* samples were considered (R^2^ = 0.321, F = 8.035, *p* = 0.001; 999 permutations; Table S3). Multivariate dispersion also differed between developmental stages (PERMDISP, *p* = 0.022); nevertheless, young and mature trees were clearly separated along the first PCoA axis, and ANOSIM supported a difference in composition (R = 0.882, *p* = 0.001) (Figure 2A). In the PCoA based on Bray–Curtis dissimilarities, the first axis explained 33.5% of the variation (Figure 2A). To further disentangle developmental-stage and locality effects, two complementary analyses were performed. When the analysis was restricted to Morungava, the only locality where both young (*n* = 11) and mature (*n* = 2) trees were sampled, developmental stage remained significantly associated with bacterial community composition (R^2^ = 0.298, F = 4.672, *p* = 0.013; 999 permutations; PERMDISP, *p* = 0.343). Conversely, when only mature trees were considered (Morungava, *n* = 2; Porto Alegre, *n* = 6), locality was also significantly associated with community composition, with locality explaining 24.8% of the observed variation (R^2^ = 0.248, F = 1.977, *p* = 0.045; 999 permutations; PERMDISP, *p* = 0.108; Table S3). Together, these analyses indicated that both developmental stage and sampling locality contributed to variation in bacterial community composition. Importantly, although locality contributed to variation in bacterial community composition, the effect of developmental stage remained significant when the analysis was restricted to Morungava, where young and mature trees were sampled within the same locality. Therefore, subsequent analyses focused on developmental stage to further characterize differences in bacterial community composition and identify taxa and functional features associated with tree developmental stage.

**Figure 3:**
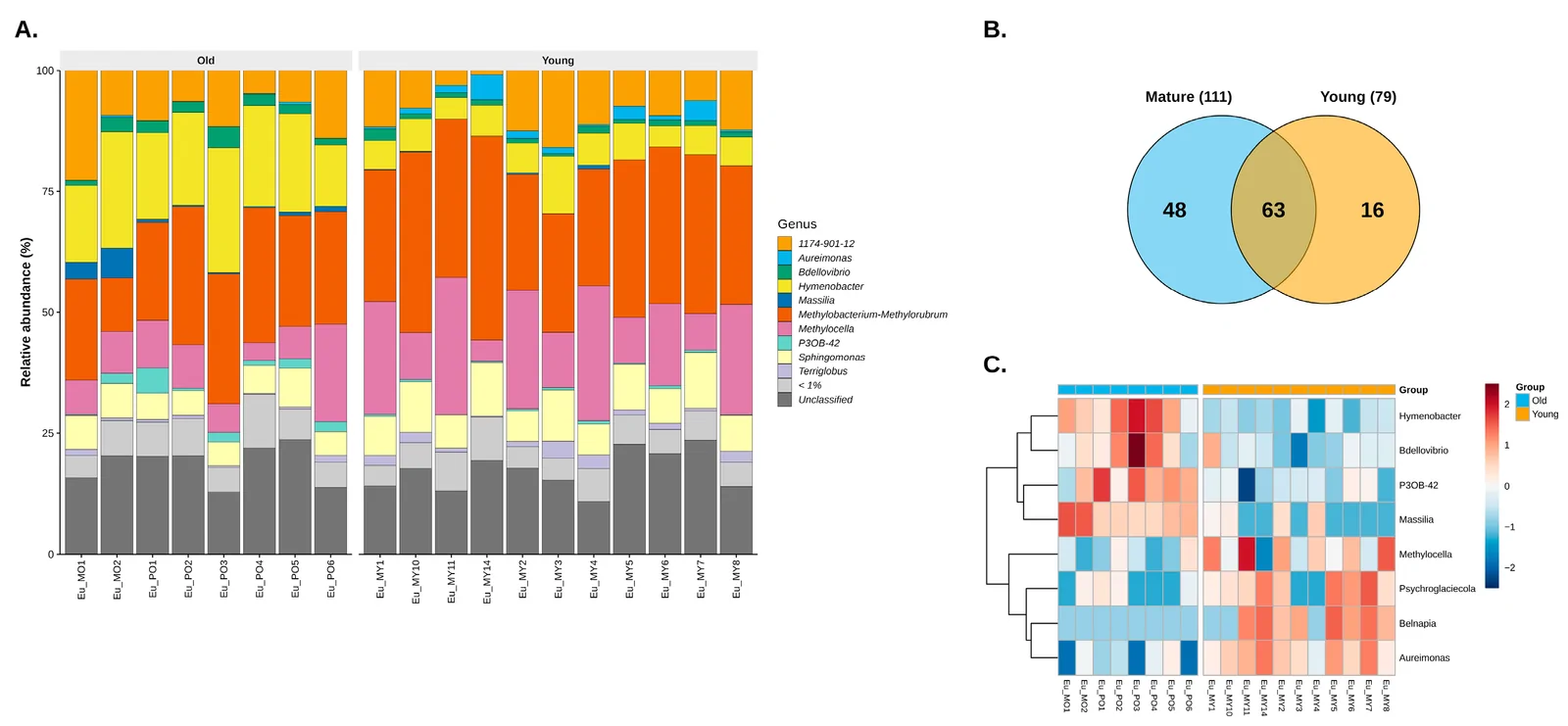
Taxonomic composition and differentially abundant bacterial genera in the phyllosphere of *Eugenia uniflora* at two developmental stages. (**A**) Relative abundance of the most abundant bacterial genera in mature and young trees; genera with a mean relative abundance below 1% in both groups were grouped as “<1%”, and reads not assigned to a genus are shown as “Unclassified”. (**B**) Venn diagram showing the number of shared and unique bacterial genera between mature and young trees based on genus presence/absence. (**C**) Heatmap of bacterial genera that differed significantly in abundance between developmental stages, as identified using edgeR (FDR < 0.05). Colors represent standardized log-transformed counts per million (Z-score), with blue indicating lower and red indicating higher relative abundance across samples.

Following taxonomic classification, the bacterial profiles across *Eugenia uniflora* (pitanga) samples were examined. At the phylum level, the most dominant groups identified were Proteobacteria (77.1%), Bacteroidota (14.7%), and Actinobacteriota (2.9%) (Figure S1A). At the family level, *Beijerinckiaceae* (53.2%), *Sphingomonadaceae* (18.4%), and *Hymenobacteraceae* (12.0%) were predominant (Figure S1B). At the genus level, the most abundant taxa included *Methylobacterium-Methylorubrum* (27.4%), *Methylocella* (13.6%), *Hymenobacter* (12.0%), 1174-901-12 (9.7%), and *Sphingomonas* (7.6%), whereas 17.8% of the reads could not be assigned to a genus (mean relative abundance over total reads; Figure 3A, Table S4).

A Venn diagram (Figure 3B) showed that young and mature trees shared 63 bacterial genera, whereas 48 and 16 genera were detected only in mature and young trees, respectively (Table S5). To move beyond presence/absence data and consider relative abundance, a differential abundance analysis was conducted using edgeR. This analysis revealed that specific bacterial genera were significantly associated with tree age. *Hymenobacter*, a member of the Myxococcaceae family (P3OB-42), and *Massilia* were more abundant in mature trees, whereas *Belnapia* and *Aureimonas* were more abundant in young trees (FDR < 0.01). *Bdellovibrio* (mature trees) and *Psychroglaciecola* and *Methylocella* (young trees) also differed between developmental stages, although close to the significance threshold (FDR = 0.046–0.047) (Figure 3C, Table S6).

**Figure 4:**
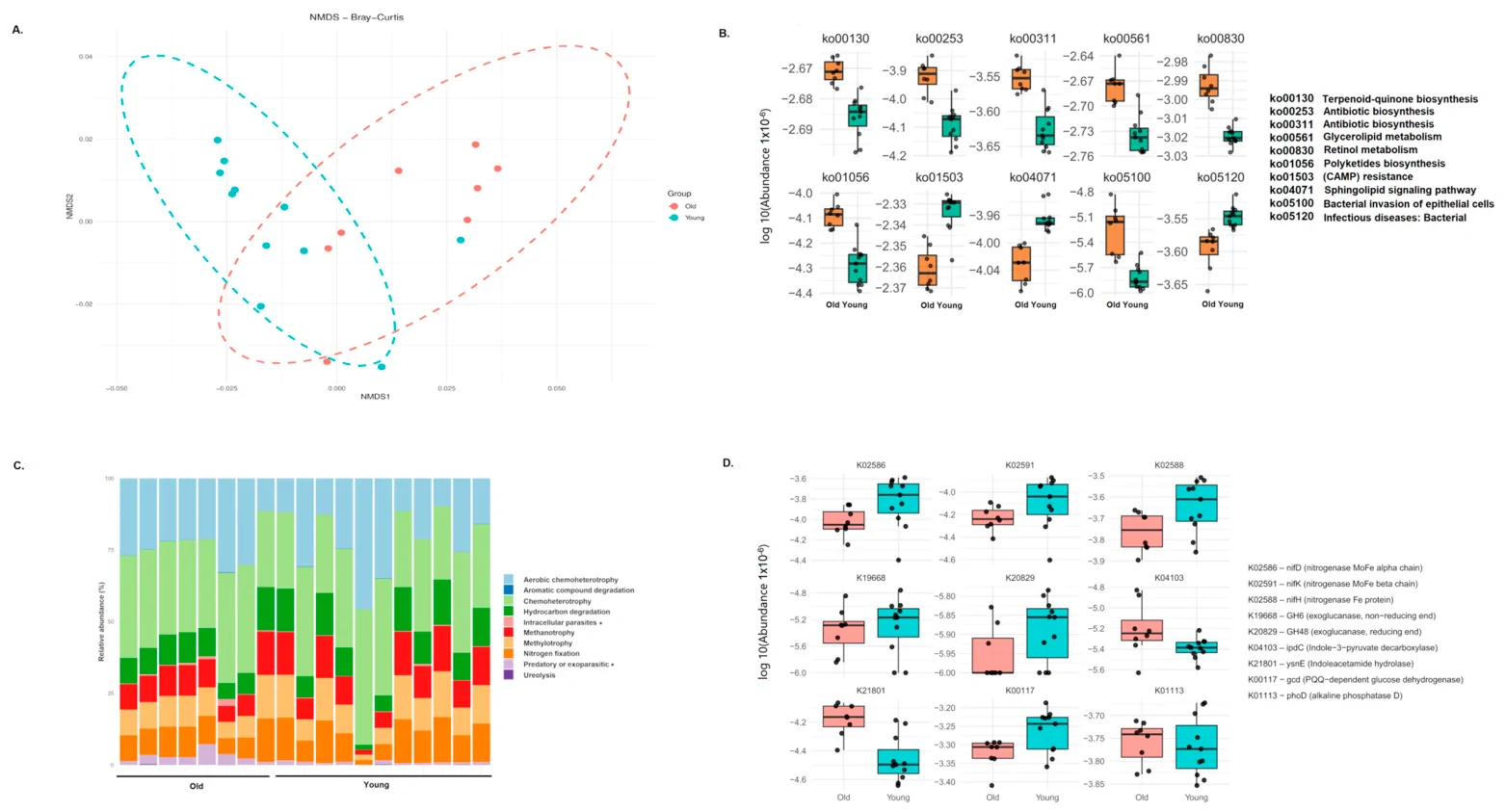
Functional potential profiling of the *Eugenia uniflora* phyllosphere bacterial community at two developmental stages. (**A**) Non-metric multidimensional scaling (NMDS) ordination of predicted KEGG functional profiles based on Bray–Curtis dissimilarities, comparing young and mature *Eugenia uniflora* trees. Ellipses represent 95% confidence intervals. Differences in functional community composition were assessed using PERMANOVA (F = 6.591, R^2^ = 0.279, *p* = 0.002, 999 permutations), NMDS stress = 0.078. (**B**) Box plots showing predicted KEGG pathways that differed significantly in relative abundance between mature and young trees (FDR < 0.05). (**C**) Relative abundance of top 10 predicted bacterial ecological functions inferred using FAPROTAX. Asterisks indicate predicted functions that differ significantly between mature and young trees. (**D**) Box plots showing the predicted abundance of selected plant growth-promoting (PGP)-associated functional genes that differed significantly between mature and young trees (FDR < 0.05). Each point represents an individual sample.

To assess the predicted metabolic and ecological functional potential of the leaf microbiome, functional categories were inferred from 16S rRNA gene sequences using Tax4Fun. This functional profiling indicated that developmental stage was associated with differences in the predicted functional potential of the bacterial community, as shown by NMDS based on Bray–Curtis dissimilarity (*p* < 0.01, Adonis test) of KEGG Orthology (KO) categories (Figure 4A, Table S7). EdgeR identified predicted functional categories with differential relative abundance between groups (Figure 4B). Mature trees showed higher relative abundance of predicted pathways associated with terpenoid-quinone (ko00130), antibiotic (ko00253, ko00311), glycerolipid (ko00561), retinol (ko00830), and polyketide (ko01056) biosynthesis, as well as bacterial invasion of epithelial cells (ko05100), whereas young trees showed higher predicted relative abundance of cationic antimicrobial peptide (CAMP) resistance (ko01503), sphingolipid signaling (ko04071) and infectious disease-associated pathways (ko05120) (Figure 4B). These results indicate that leaf microbiome functional potential shifts with plant age, with mature-tree samples exhibiting a higher predicted contribution of pathways related to secondary metabolism, whereas pathways associated with signaling and disease-related categories were more strongly represented in young-tree samples. Predicted functional potential across all samples included methanotrophy, methylotrophy, nitrogen fixation, aerobic chemoheterotrophy, hydrocarbon degradation, predatory or exoparasitic behavior, and general chemoheterotrophy (Figure 4C; Table S8). DESeq2 analysis revealed that predatory or exoparasitic behavior and intracellular parasitism were predicted at higher relative abundance in mature-tree samples. These differences indicate variation in predicted ecological functional potential between developmental stages, but do not provide direct evidence that these ecological interactions occur in the *E. uniflora* phyllosphere.

Likewise, the predicted abundance of PGP-associated functional categories varied according to developmental stage (Figure 4D). The predicted functional profiles included KEGG Orthologs associated with the nitrogenase complex such as nifH (K02588), nifD (K02586), and nifK (K02591), with a trend toward higher predicted abundance in the phyllosphere of young trees. KEGG Orthologs associated with auxin biosynthesis, including ipdC (K04103) and ysnE (K21801), were predicted in both groups, with ipdC showing relatively higher predicted abundance in mature-tree samples. Similarly, KEGG Orthologs associated with cellulolytic activity, including GH6 (K19668) and GH48 (K20829), and phosphate solubilization, including gcd (K00117) and phoD (K01113), were broadly predicted across samples, although gcd showed higher predicted abundance in young-tree samples. These patterns suggest differences in the predicted functional potential of the phyllosphere bacterial communities associated with plant developmental stage. However, these predictions do not demonstrate the presence, expression, or activity of the corresponding genes and require experimental validation.

### 2.2. Co-Occurrence Network Analysis Reveals Differences in Bacterial Co-Occurrence Patterns Between Developmental Stages

**Figure 5:**
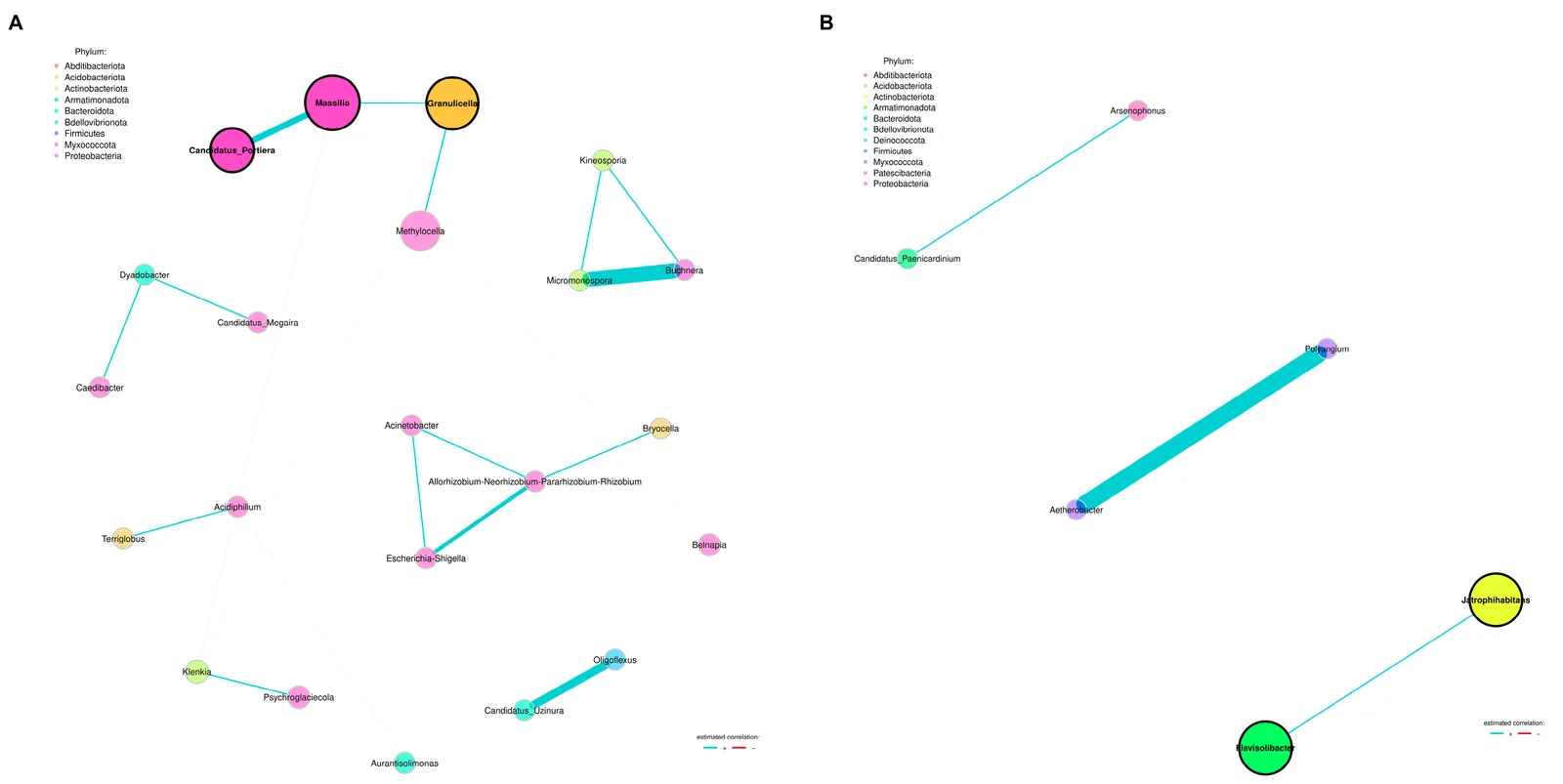
Co-occurrence networks of the phyllosphere bacterial community in *Eugenia uniflora* at two developmental stages. Network visualizations based on Spearman correlations at the genus level for (**A**) young plants and (**B**) mature plants. Nodes represent bacterial genera, and edges indicate statistically significant correlations (|ρ| ≥ 0.3, FDR-adjusted *p* < 0.05, Benjamini–Hochberg adaptive procedure). Node size is proportional to eigenvector centrality, reflecting the relative influence of each genus within the network. Node colors denote phylum-level taxonomic affiliation, as shown in the legend. Positive correlations are represented by dark turquoise edges, while negative correlations are shown as red edges; edge thickness and color intensity are proportional to the absolute correlation coefficient |ρ|. Only genera that exhibited significant co-occurrence patterns are displayed; taxa with stable, non-varying abundances across samples were not captured in the networks.

To investigate patterns of association among bacterial taxa, co-occurrence networks were constructed based on Spearman correlations at the bacterial genus level for young and mature *Eugenia uniflora* plants. The network associated with young plants comprised 22 genera, including *Terriglobus*, *Acidiphilium*, *Belnapia*, *Massilia*, *Granulicella*, *Candidatus Portiera*, *Kineosporia*, *Methylocella*, *Dyadobacter*, *Buchnera*, *Micromonospora*, *Candidatus Megaira*, *Caedibacter*, *Acinetobacter*, *Bryocella*, *Allorhizobium–Neorhizobium–Pararhizobium–Rhizobium*, *Escherichia–Shigella*, *Oligoflexus*, *Klenkia*, *Psychroglaciecola*, *Candidatus Uzinura*, and *Aurantisolimonas* (Figure 5). The genera *Massilia*, *Candidatus Portiera*, and *Granulicella* exhibited the highest eigenvector centrality and therefore occupied central positions within the network, contributing to a greater number or strength of associations with other taxa.

The young-plant network contained both negative and positive associations. Negative associations were observed between *Massilia* and *Klenkia*, *Massilia* and *Belnapia*, *Granulicella* and *Klenkia*, *Acidiphilium* and *Aurantisolimonas*, and *Candidatus Portiera* and *Belnapia*. Positive associations included *Granulicella* with *Methylocella*; *Kineosporia* with both *Micromonospora* and *Buchnera*; *Acinetobacter* with *Allorhizobium–Neorhizobium–Pararhizobium–Rhizobium*, *Escherichia–Shigella*, and *Bryocella*; *Terriglobus* with *Acidiphilium*; *Klenkia* with *Psychroglaciecola*; *Caedibacter* with *Dyadobacter* and *Candidatus Megaira*; and *Candidatus Uzinura* with *Oligoflexus*. *Belnapia*, which was more abundant in young plants, was represented in the network together with *Massilia*, which was more abundant in mature plants. This pattern indicates that the associations inferred from co-occurrence analysis were not restricted to taxa showing similar abundance patterns between developmental stages. Instead, they reflected covariation in taxon abundances across individual samples, which may result from shared responses to host or environmental conditions. Because correlation-based networks do not establish direct ecological interactions, these associations should be interpreted as patterns of co-occurrence rather than evidence of direct microbial interactions.

In contrast, the network associated with mature plants was considerably smaller, comprising only six genera: *Candidatus Paenicardinium*, *Arsenophonus*, *Polyangium*, *Aetherobacter*, *Jatrophihabitans*, and *Flavisolibacter*. Among these, *Flavisolibacter* and *Jatrophihabitans* exhibited the highest eigenvector centrality and therefore occupied central positions within the network. Significant positive correlations were detected between *Candidatus Paenicardinium* and *Arsenophonus*, *Aetherobacter* and *Polyangium*, and *Flavisolibacter* and *Jatrophihabitans*.

Notably, the genera that were most abundant in mature plants, including *Massilia* and *Hymenobacter*, as well as taxa detected in all mature samples, were absent from the inferred co-occurrence network. This absence does not indicate reduced ecological importance. Rather, these taxa exhibited very low abundance variability among mature samples (coefficient of variation < 0.2), which reduced the opportunity to detect significant correlations with other taxa. Consequently, the network primarily represented taxa whose abundances varied sufficiently across samples to support significant associations, whereas dominant taxa with relatively consistent abundances were not represented in the inferred network. These findings are consistent with the presence of dominant and relatively stable taxa within the mature phyllosphere, while the co-occurrence network captured associations among taxa exhibiting greater between-sample variability.

The identification of *Jatrophihabitans* as a central taxon in the mature-plant network is noteworthy. This genus tended to be more abundant in young plants (FDR = 0.09), was detected in only a subset of mature individuals, and displayed relatively high variability among samples. This pattern suggests that *Jatrophihabitans* may be associated with a subset of mature plants in which variation in its abundance was coordinated with that of other taxa. Its positive association with *Flavisolibacter* may reflect shared responses to host or environmental conditions, although the correlation alone does not establish a direct ecological interaction or a shared niche.

Overall, the co-occurrence networks revealed differences in the patterns of taxon associations between developmental stages. The young-plant network contained a greater number of positive and negative associations, whereas the mature-plant network was smaller and comprised fewer inferred associations.

### 2.3. Comparison of Bacterial Diversity and Shared Taxa Between Eugenia uniflora and Other Myrtaceae Species

The bacterial community associated with the 21 mature Myrtaceae trees (8 *E. uniflora* and 13 trees of eight other species) comprised 1790 ASVs (Amplicon Sequence Variants; Table S2). Cross-host comparisons were conducted at both the species and genus levels, using individual tree samples as the statistical units. Sample sizes were unbalanced among the host groups (Table S9). At the species level, Kruskal–Wallis tests did not detect significant differences among hosts for Observed richness (χ^2^ = 12.90, df = 8, *p* = 0.115), Chao1 (χ^2^ = 12.77, df = 8, *p* = 0.120), ACE (χ^2^ = 12.77, df = 8, *p* = 0.120), Shannon diversity (χ^2^ = 14.52, df = 8, *p* = 0.069), or Simpson diversity (χ^2^ = 13.89, df = 8, *p* = 0.085) (Figure 1B). At the genus level, richness estimates did not differ (Observed, χ^2^ = 6.43, df = 3, *p* = 0.093; Chao1 and ACE, χ^2^ = 6.29, df = 3, *p* = 0.098), whereas Shannon (χ^2^ = 8.08, df = 3, *p* = 0.045) and Simpson diversity (χ^2^ = 8.84, df = 3, *p* = 0.031) showed a significant overall effect; however, no pairwise comparison remained significant after adjustment for multiple comparisons (Table S9). Bacterial community composition differed among host species (PERMANOVA, R^2^ = 0.607, F = 2.31, *p* = 0.001) and among host genera (R^2^ = 0.237, F = 1.76, *p* = 0.002; PERMDISP, *p* = 0.265) (Figure 2B). Homogeneity of dispersion could not be tested at the species level because three species were represented by a single tree.

Taxonomic profiling revealed that the dominant bacterial phyla across all samples were Proteobacteria (66.7%), Bacteroidota (22.7%), and Actinobacteriota (3.8%) (Figure S2A). At the family level, the most abundant groups were *Beijerinckiaceae* (32.1%), *Sphingomonadaceae* (23.6%), and *Hymenobacteraceae* (20.4%) (Figure S2B). Comparative analysis further revealed a set of bacterial genera shared between *Eugenia uniflora* and the other Myrtaceae hosts evaluated, including taxa detected across all sampled host groups. These shared taxa provide evidence of conserved bacterial members among the Myrtaceae hosts examined in this study.

**Figure 6:**
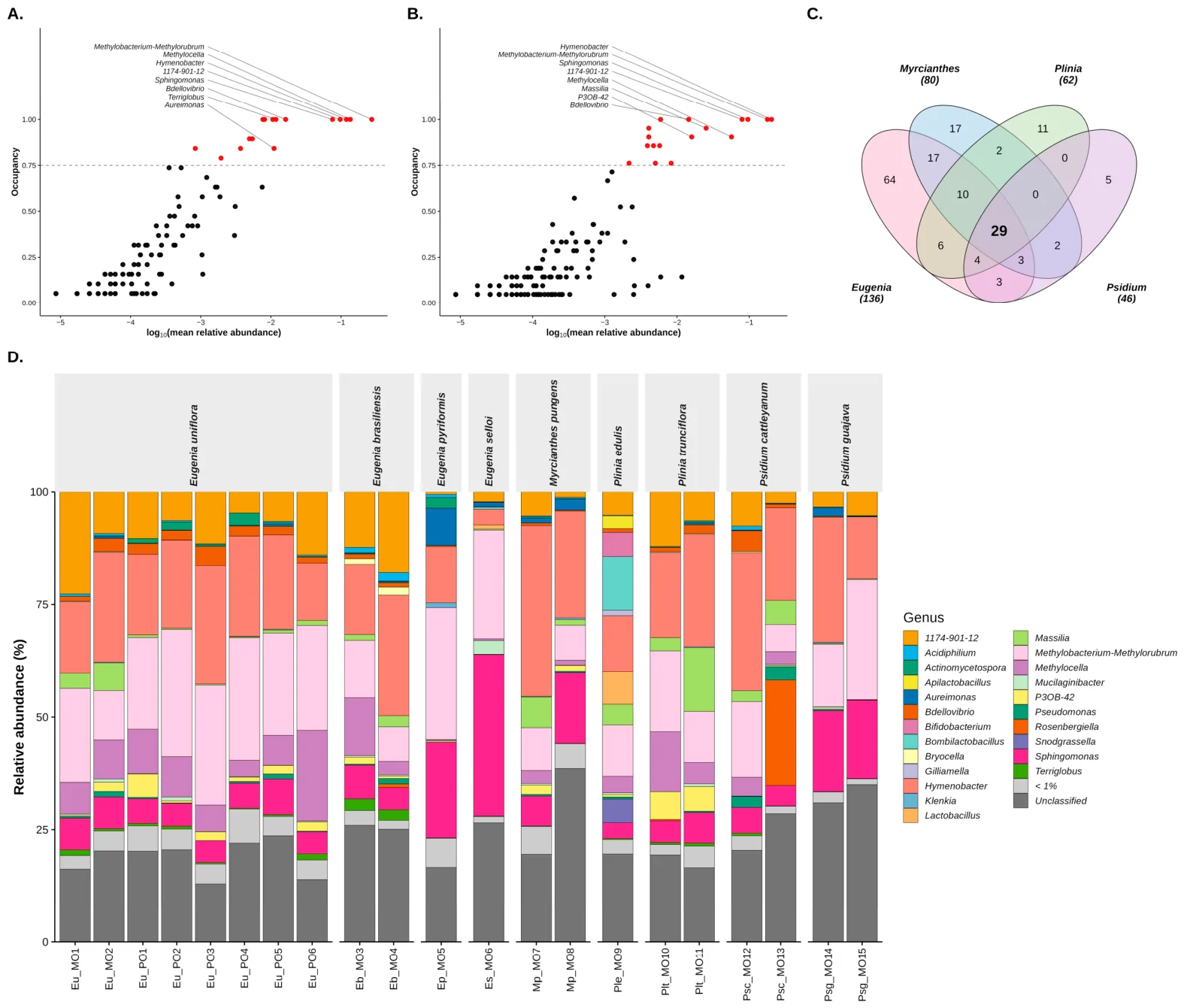
Core microbiome of *Eugenia uniflora* and other Myrtaceae species. (**A**) Core microbiome of *E. uniflora* showing bacterial genera occupancy versus log-transformed mean relative abundance. Genera detected in at least 75% of samples were defined as core taxa and are highlighted in red; the eight most abundant core genera are labeled. In (**A**,**B**), the dashed line indicates the 75% occupancy threshold. (**B**) Core microbiome of the 21 mature Myrtaceae trees included in this study, showing bacterial genera occupancy versus log-transformed mean relative abundance. Core taxa (≥75% sample occupancy) are highlighted in red, and the eight most abundant core genera are labeled. (**C**) Venn diagram showing the number of unique and shared bacterial genera among the Myrtaceae genera (*Eugenia*, *Myrcianthes*, *Plinia*, and *Psidium*). A bacterial genus was considered present in a plant genus when detected in at least one sample. (**D**) Relative abundance of the most abundant bacterial genera in the 21 mature trees, grouped by host species; genera with a mean relative abundance below 1% in all species were grouped as “<1%”, and reads not assigned to a genus are shown as “Unclassified”.

A total of 16 bacterial genera were identified as part of the core microbiome of *E. uniflora*, defined as genera detected in at least 75% of samples (Figure 6A, Table S10). Extending this analysis to the other Myrtaceae hosts revealed several bacterial genera shared across the sampled hosts (Figure 6B, Table S11). The recurrence of these taxa across different Myrtaceae genera indicates that a subset of bacterial taxa is conserved among the hosts examined in this study, supporting the presence of shared bacterial members within the Myrtaceae phyllosphere. To further assess the overlap of bacterial taxa among host species, a Venn diagram was generated (Figure 6C), showing that 29 bacterial genera were detected in all four host genera. In the mature-tree dataset, 17 genera met the predefined core criterion [18], being detected in at least 75% of samples (in at least 16 of the 21 trees; Table 1 and Table S11). Notably, six genera, *Hymenobacter*, Methylobacterium*–Methylorubrum*, *Sphingomonas*, 1174-901-12, *Bdellovibrio*, and *Terriglobus*, were detected in 100% of the samples (Table 1, Figure 6D). The recurrent occurrence of these taxa across different Myrtaceae species identifies a set of bacterial genera consistently associated with the phyllosphere of the hosts examined in this study, supporting the presence of conserved bacterial taxa among the sampled Myrtaceae.

Additionally, approximately 0.73% of the bacterial sequences remained unclassified at the phylum level, indicating the presence of potentially novel or poorly characterized taxa within these plant-associated communities.

Functional potential prediction of the bacterial community associated with Myrtaceae hosts revealed that the predominant predicted functions included aerobic and general chemoheterotrophy, fermentation, hydrocarbon degradation, methanotrophy, methylotrophy, nitrogen fixation, and ureolysis, all of which are potentially important for plant growth and ecosystem functioning (Figure S3; Table S12). These predictions indicate the potential for diverse metabolic processes within the Myrtaceae-associated bacterial communities, including carbon and nitrogen transformations.

### 2.4. Conserved Bacterial Interaction Patterns Among the Sampled Myrtaceae Hosts

**Figure 7:**
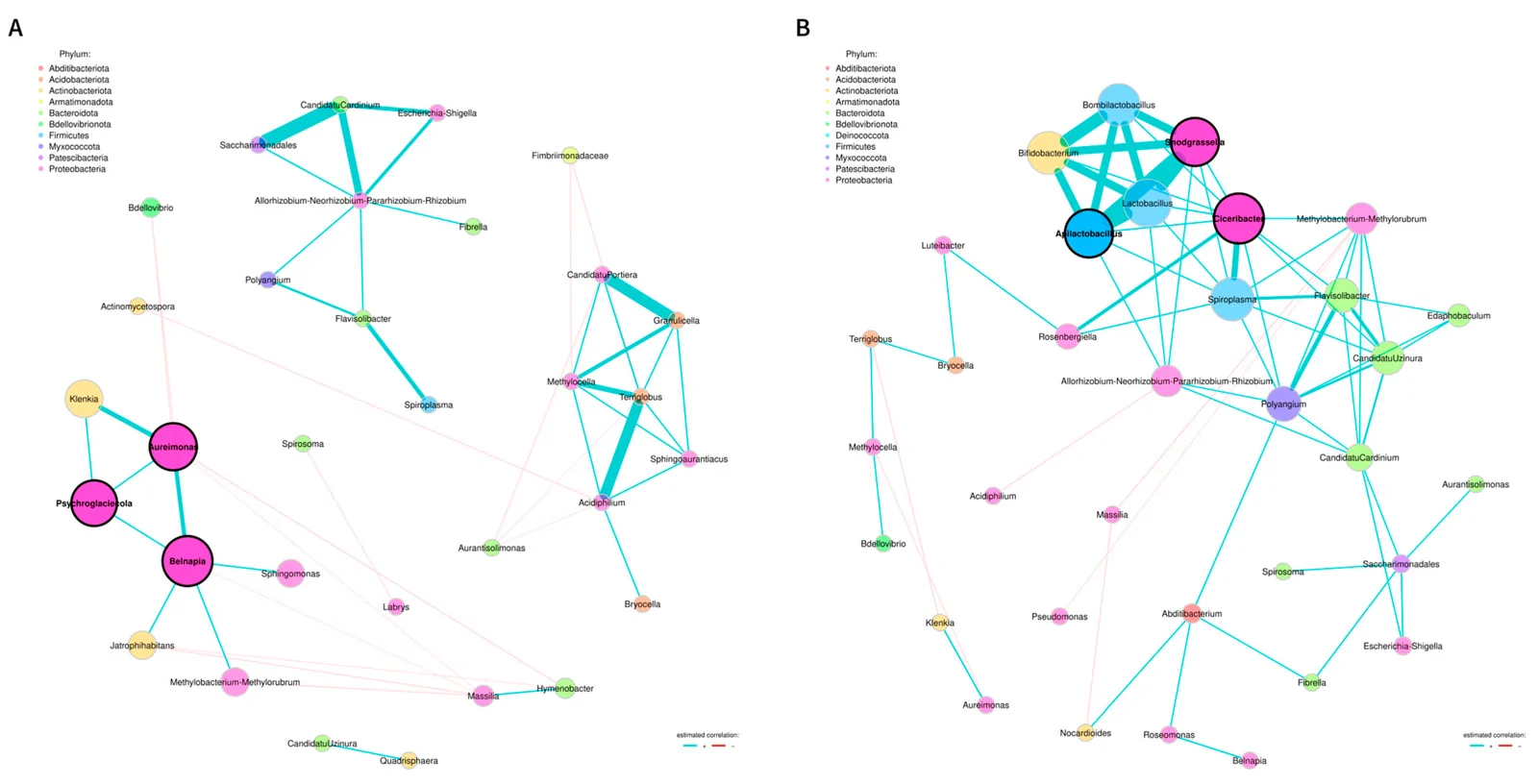
Co-occurrence networks of bacterial genera associated with Myrtaceae. (**A**) Network for *Eugenia uniflora*. (**B**) Network for the Myrtaceae dataset (multiple host genera). Nodes represent bacterial genera, colored by phylum. Node size is proportional to eigenvector centrality. Edges indicate significant Spearman correlations (*p* < 0.05); turquoise edges represent positive associations, whereas red edges represent negative associations; edge thickness and color intensity are proportional to the absolute correlation coefficient |ρ|. A conserved core of hub genera is evident in both panels, although the most influential hub differs (*Belnapia* in panel (**A**), *Ciceribacter* in panel (**B**)).

At the genus level, co-occurrence network analysis revealed several highly connected taxa shared between *Eugenia uniflora* and Myrtaceae network (Figure 7). Genera including *Ciceribacter*, *Spiroplasma*, *Polyangium*, *Methylobacterium–Methylorubrum*, *Lactobacillus*, *Snodgrassella*, and *Apilactobacillus* consistently exhibited high degree, closeness, and eigenvector centralities, indicating that they occupied central positions in the interaction networks. Although the identity of the principal hub differed between datasets, with *Belnapia* representing the most influential node in *E. uniflora* and *Ciceribacter* in the Myrtaceae network, the recurrence of several highly connected genera suggests that the overall organization of bacterial interactions is partially shared across the hosts examined. Several co-occurrence patterns were also recurrent between the two networks. Positive associations between *Aureimonas* and *Klenkia*, *Methylocella* and *Terriglobus*, and *Terriglobus* and *Bryocella* were detected in both networks, indicating recurrent associations among these taxa. Likewise, the recurrent positive association between *Spiroplasma* and *Flavisolibacter* suggests that this relationship is shared between the networks. In contrast, the negative association between *Methylobacterium–Methylorubrum* and *Massilia* was detected in both networks, potentially reflecting differences in their co-occurrence patterns. Overall, the genus-level network analysis indicates that, despite host-specific differences in the identity of the dominant hubs, the Myrtaceae phyllosphere exhibits shared features, including recurrent, highly connected bacterial genera. These conserved interaction patterns suggest that the sampled Myrtaceae hosts harbor bacterial communities with similar organizational properties while maintaining species-specific variation in network structure.

## 3. Discussion

### 3.1. Eugenia uniflora Developmental Stage Is Associated with Phyllosphere Bacterial Community Assembly and Predicted Functional Potential

The phyllosphere of mature *E. uniflora* trees exhibited higher Shannon and Simpson diversity than that of young trees, while beta-diversity analyses revealed distinct community compositions between developmental stages. These findings support the hypothesis that host developmental stage is an important factor associated with phyllosphere microbiome assembly and are consistent with previous studies demonstrating that plant ontogeny strongly influences microbial recruitment and community structure in diverse plant species [16,29]. However, sampling locality was also associated with variation in bacterial diversity and community composition, highlighting the contribution of local environmental conditions to phyllosphere microbiome structure. Importantly, the association with developmental stage remained evident when young and mature trees were compared within the same locality, suggesting that developmental stage contributes to bacterial community variation beyond the effect of sampling location.

Because epiphytic and endophytic microorganisms were not experimentally separated, the bacterial profiles observed here represent the overall leaf-associated bacterial community. Therefore, the differences observed between developmental stages reflect changes in the total bacterial community associated with leaves rather than changes attributable specifically to either epiphytic or endophytic fractions. The observed differences likely result from age-dependent physiological and biochemical changes occurring during plant development. As leaves mature, they experience modifications in cuticle structure, nutrient availability, surface morphology, and the accumulation of secondary metabolites, including flavonoids and terpenoids, which are characteristic of Myrtaceae [30,31]. These changes alter the physicochemical environment of the leaf surface and create new ecological niches that selectively favor microorganisms possessing traits that enable them to tolerate antimicrobial compounds, withstand desiccation and ultraviolet radiation, and efficiently utilize plant-derived carbon sources [8]. Consequently, microbiome assembly is probably driven not only by prolonged exposure of mature trees to environmental microbial pools but also by developmental changes in host physiology that actively shape microbial recruitment [17].

The predicted functional profiles further support this interpretation. Bacterial communities associated with mature trees showed greater predicted representation of pathways related to secondary metabolism, whereas those associated with young trees showed greater predicted representation of pathways related to microbial interactions and signaling. From an ecological perspective, the greater bacterial diversity observed in mature trees may also enhance community stability through increased functional redundancy, thereby improving resilience against environmental disturbances and pathogen invasion. Such diversity may contribute to nutrient cycling, stress tolerance, and disease suppression, reinforcing the concept that plants and their associated microbiota function as an integrated ecological unit, or holobiont [32].

However, these functional profiles should be interpreted with caution because they were inferred from 16S rRNA gene sequencing rather than obtained from direct functional measurements. Tax4Fun-based predictions provide an estimate of the functional potential of the bacterial community based on taxonomic assignments and reference genomes, but they do not demonstrate the presence, expression, or activity of the predicted genes or pathways. Therefore, the differences observed between developmental stages should be considered hypotheses regarding potential functional differences rather than evidence of actual metabolic or ecological activities in the phyllosphere. These predictions are consistent with the hypothesis that developmental changes in host physiology may influence not only the taxonomic composition but also the functional potential of phyllosphere microbial communities. Further validation using approaches such as shotgun metagenomics, metatranscriptomics, or targeted functional assays would be necessary to confirm these predicted functional differences.

### 3.2. Conserved Bacterial Taxa Suggest Potential Ecological Filtering Among Selected Myrtaceae Species

Beyond developmental effects, comparative analyses identified a set of bacterial genera consistently associated with *E. uniflora* and other Myrtaceae taxa represented by samples from four genera. Proteobacteria, Bacteroidota, and Actinobacteriota dominated all phyllosphere communities, while *Methylobacterium–Methylorubrum*, *Hymenobacter*, *Sphingomonas*, 1174-901-12, *Bdellovibrio*, and *Terriglobus* were detected in all 21 mature trees. The repeated occurrence of these taxa across phylogenetically related hosts suggests the existence of conserved microbial associations within Myrtaceae.

Rather than necessarily reflecting strict co-evolutionary relationships, these conserved associations are likely driven by ecological filtering imposed by shared host characteristics. Members of the Myrtaceae possess several common leaf traits [33], including thick cuticles, wax-rich surfaces [30], and high concentrations of essential oils, flavonoids, and terpenoids, all of which create a chemically and physically selective environment for microbial colonization [31,34]. Under these conditions, bacterial taxa possessing adaptations such as resistance to ultraviolet radiation and desiccation, efficient utilization of plant-derived carbon compounds, biofilm formation, or tolerance to antimicrobial secondary metabolites are more likely to establish stable populations across multiple hosts. Consequently, the conserved bacterial genera identified here probably reflect functional convergence toward a common phyllosphere niche rather than host specificity alone.

Interestingly, five of the six bacterial genera consistently detected across all sampled Myrtaceae exhibit ecological traits well suited for life on leaf surfaces. Species of *Methylobacterium–Methylorubrum* efficiently utilize methanol released during plant metabolism and have been widely reported as dominant members of phyllosphere communities, where they promote plant growth and improve tolerance to abiotic stress. Likewise, *Sphingomonas* and *Hymenobacter* are recognized for their remarkable resistance to ultraviolet radiation, oxidative stress, and desiccation, traits that facilitate persistence on exposed leaf surfaces. In addition, *Sphingomonas* has frequently been associated with pathogen suppression and degradation of aromatic compounds, whereas *Hymenobacter* may contribute to stress tolerance and modulation of plant metabolism [35,36,37]. *Bdellovibrio*, a predatory bacterium, may contribute to microbiome stability by regulating populations of Gram-negative bacteria, thereby potentially suppressing pathogens and maintaining community balance [38,39]. Although *Terriglobus* is traditionally regarded as a soil-associated genus, its consistent detection across all sampled hosts suggests that it may possess broader ecological versatility than previously recognized, potentially contributing to carbon cycling and nutrient turnover within the phyllosphere [40,41,42].

The identification of these conserved bacterial taxa contributes to the understanding of microbiome assembly within Myrtaceae and provides a valuable starting point for future functional studies. Because these genera occur consistently across multiple hosts despite differences in host species, they represent promising candidates for investigating stable plant–microbe interactions and their potential applications in plant growth promotion, biological control, and microbiome engineering. Nevertheless, the ecological functions proposed here are inferred from previous studies and predictive functional analyses and therefore require experimental validation in *E. uniflora* and other Myrtaceae species.

The present study also has several limitations that should be considered when interpreting these findings. First, the use of 16S rRNA gene amplicon sequencing limited taxonomic resolution to approximately the genus level and prevented direct characterization of microbial functional genes. Second, although both sampling sites are located within the same climatic region and samples were collected using identical protocols, young trees were available only at one location, preventing complete separation of developmental and environmental effects. Future studies employing balanced sampling across multiple sites will help clarify the relative contributions of host age and local environmental conditions to microbiome assembly. Third, the comparative analysis across Myrtaceae was constrained by the limited and unbalanced sampling among host species, including species represented by a single individual, which limits statistical power and precludes broad conclusions about conserved bacterial taxa across the Myrtaceae family. Therefore, the conserved taxa identified here should be interpreted as patterns observed among the Myrtaceae hosts sampled in this study, rather than as evidence of family-wide conservation. Fourth, sampling was restricted to two developmental stages and did not evaluate seasonal or long-term temporal dynamics. Finally, only the phyllosphere was investigated, whereas interactions with belowground microbial communities were beyond the scope of this study. Integrating shotgun metagenomics, metabolomics, microbial isolation, and functional assays with broader spatial and temporal sampling will be essential to elucidate the mechanisms underlying these conserved plant–microbe associations and to validate their ecological and biotechnological significance.

### 3.3. Developmental Changes in Bacterial Co-Occurrence Networks of Eugenia uniflora and Shared Patterns Among Selected Myrtaceae Species

The contrasting network structures between young and mature *E. uniflora* trees suggest that plant development may influence the organization of bacterial communities in the phyllosphere. The greater connectivity observed in young plants may reflect a more dynamic community assembly associated with the changing physiological conditions of developing leaves, whereas the simpler network in mature trees may be consistent with a more stable community structure. However, because co-occurrence networks capture statistical associations rather than direct biological interactions, these patterns cannot distinguish direct microbial interactions from shared responses to host or environmental conditions [2,4,43].

Highly connected genera such as *Massilia*, *Granulicella*, and *Candidatus Portiera* in young plants may represent candidate taxa for further investigation of bacterial community organization during early plant development. Conversely, the absence of abundant genera such as *Massilia* and *Hymenobacter* from the mature-tree network illustrates that abundance and network centrality represent different properties of a microbial community. Their relatively consistent abundance among mature samples may have limited their contribution to correlation-based network structure, which instead captured variation among less abundant taxa.

Comparisons among the selected Myrtaceae hosts revealed recurrent co-occurrence patterns despite differences in network structure. For example, the repeated association between *Terriglobus* and *Methylocella* may reflect shared or complementary resource-use strategies. *Terriglobus* has been associated with the degradation of complex plant-derived polysaccharides [44], whereas *Methylocella* can utilize methane, methanol, and other carbon substrates [45]. Nevertheless, these associations should be regarded as hypotheses rather than evidence of direct ecological interactions.

The identity of highly connected taxa also differed among hosts, with *Belnapia* prominent in *E. uniflora* and *Ciceribacter* in the multi-host network. This variation suggests that similar patterns of bacterial community organization may occur through different taxa, potentially reflecting host-specific and environmental influences. Overall, the results support a combination of development-associated changes in network organization and recurring, but not necessarily taxonomically conserved, bacterial associations among the selected Myrtaceae hosts.

## 4. Materials and Methods

### 4.1. Plant Sample Collection

To characterize the bacterial communities associated with *Eugenia uniflora* and to assess differences associated with plant developmental stage, 19 individual trees were sampled from apparently healthy plants, including eleven young trees (2 years old, approximately 1 m in height) and eight mature trees (approximately 15–25 years old). Fully expanded, apparently healthy leaves were collected from multiple locations across the canopy of each tree to capture within-plant variation in microbial communities. Thirteen samples (eleven young and two mature trees) were collected at Margis Farm, located in the Morungava district of Gravataí (29°51′52.7″ S, 50°53′49.8″ W), where both young and mature *E. uniflora* trees were available. The remaining six samples were collected at the Campus do Vale of the Federal University of Rio Grande do Sul (UFRGS), in Porto Alegre (30°04′23″ S, 51°07′33″ W), where only mature trees were present. Both sampling sites are located in Rio Grande do Sul, southern Brazil, approximately 48 km apart, and experience a humid subtropical climate (Cfa, Köppen classification), with similar temperature and precipitation regimes. All samples were collected during the same sampling period using identical collection procedures to minimize environmental and methodological variation (Table 2).

To compare the phyllosphere microbiomes of pitanga and related Myrtaceae trees, leaf samples were collected from 21 mature trees representing four genera. The number of individual trees sampled varied among genera: *Eugenia* (*n* = 12), *Myrcianthes* (*n* = 2), *Plinia* (*n* = 3), and *Psidium* (*n* = 4). For each tree, leaves were collected from multiple locations across the canopy to capture within-plant variation in microbial communities. In total, nine Myrtaceae species were included in the analysis (Table 2): *Eugenia uniflora* (pitanga, *n* = 8; two trees from Morungava and six from Porto Alegre), *Eugenia brasiliensis* (grumixama, *n* = 2), *Eugenia pyriformis* (uvaia, *n* = 1), *Eugenia selloi* (pitangão, *n* = 1), *Myrcianthes pungens* (guabijú, *n* = 2), *Plinia trunciflora* (jaboticaba, *n* = 2), *Plinia edulis* (cambucá, *n* = 1), *Psidium guajava* (guava, *n* = 2), *Psidium cattleyanum* (araçá, *n* = 2). The trees of the eight additional Myrtaceae species were all sampled at Morungava and were located within a one-hectare area, with a minimum spacing of 50 cm between individuals. All samples were collected in November 2021, during the spring season.

The comparisons were conducted separately according to the objectives of each analysis. For diversity and community composition analyses, predicted functional profiles, and co-occurrence network analyses within *E. uniflora*, young and mature trees were compared to assess differences associated with plant developmental stage. For the comparative Myrtaceae analyses, only mature trees were considered, including samples from Morungava and Porto Alegre. Bacterial communities were compared at both the species and genus levels. At the species level, mature *E. uniflora* was compared separately with each of the other sampled mature Myrtaceae species. At the genus level, samples were compared according to their host genus, including pairwise comparisons among *Eugenia*, *Myrcianthes*, *Plinia*, and *Psidium*. The core microbiome and Venn analyses of Myrtaceae included the same 21 mature trees, whereas the core microbiome of *E. uniflora* was determined using all 19 *E. uniflora* trees.

### 4.2. Collection of Microbiome Community

Leaf material from each sample was transported to the laboratory in sterile plastic bags. To characterize the total leaf-associated bacterial community, including both epiphytic and endophytic microorganisms, leaf fragments were processed without prior surface sterilization. Leaves were cut into small pieces (~0.5 × 0.5 mm) using sterile tweezers and a scalpel on a sterilized glass Petri dish under a laminar flow hood. The leaf fragments were transferred to an Erlenmeyer flask containing 50 mL of sterile, pre-chilled PBST buffer. Samples were shaken at 180 rpm for 2 h at 4 °C. After incubation, 40 mL of the suspension was transferred to sterile Falcon tubes and centrifuged at 3000 rpm for 30 min. The resulting pellets were stored at −20 °C until DNA extraction.

### 4.3. DNA Isolation and Sequencing

Total DNA was extracted using the ZymoBIOMICS™ DNA MiniPrep Kit (Zymo Research Corporation, Irvine, CA, USA). DNA quality and concentration were assessed using agarose gel electrophoresis and a NanoDrop spectrophotometer (Thermo Fisher Scientific, Wilmington, MA, USA. The V3–V4 region of the 16S rRNA gene was amplified using the primer pair 341F and 806R [46,47]. PCR amplification was performed following the protocol described by Christoff et al. (2017) [48]. The PCR products were visualized on a 1.5% agarose gel, and samples showing a clear band between 400 and 450 bp were selected for sequencing. Amplicon library preparation, including indexing and pooling of PCR products, was performed by Neoprospecta (Florianópolis, Brazil). Sequencing was carried out on an Illumina MiSeq platform (Illumina, San Diego, CA, USA) as part of a complete NGS workflow provided by the company.

### 4.4. Sequence Analysis

Raw sequence data were first assessed for quality using FastQC (version 0.13.0), and low-quality reads (Phred score < 30) and sequences shorter than 200 nucleotides were filtered out using Trim Galore (version 2.3.0; https://github.com/FelixKrueger/TrimGalore (accessed on 1 October 2025)). To remove host-derived contamination, Bowtie2 (version 2.5.5) was used to map and filter reads corresponding to chloroplast (cp) and mitochondrial (mt) sequences by aligning against *Eugenia uniflora* cp16S rRNA and Eucalyptus grandis mt18S rRNA gene references. These organellar reads were excluded from further analysis.

Subsequent quality control and processing were performed using the DADA2 pipeline [49], as implemented in QIIME 2 (version 2024.10), which included read filtering, trimming, denoising, and chimera removal. Amplicon sequence variants (ASVs) were inferred at 100% sequence similarity. Representative sequences for each ASV were aligned using MAFFT in QIIME 2, and phylogenetic trees (unrooted and rooted) were generated with FastTree to support downstream diversity analyses.

Taxonomic classification of ASVs was performed in QIIME 2 (version 2024.10) [46] using a pre-trained Naïve Bayes classifier against the SILVA 16S rRNA database (SSU release 138.2; https://www.arb-silva.de/ (accessed on 1 October 2025)) [50,51], applying a minimum confidence threshold of 0.8. An ASV table was constructed to represent the distribution of bacterial taxa across samples, and taxonomic bar plots were generated using the ggplot2 package in R-4.6.1 for the phylum, family, and genus levels.

ASVs identified as plant-derived—classified as Cyanobacteria/Chloroplast or Proteobacteria/Mitochondria, were filtered out using QIIME2’s taxonomy-based feature table filtering option. In total, 32 libraries representing four plant genera were analyzed. After all filtering steps, sequencing depth ranged from 5530 reads (*Plinia edulis*) to 30,908 reads (*Eugenia uniflora*) per sample. Detailed information on read counts and ASV numbers per sample is provided in Supplementary Tables S1 and S2. The raw sequence data are publicly available in the GenBank database under BioProject accession number PRJNA1122548.

### 4.5. Statistical Analyses

The relative abundance of ASV was calculated after rarefying all samples to the minimum sequencing depth observed per sample. Rarefaction was performed separately for the *E. uniflora* dataset and for the mature-tree Myrtaceae dataset. Subsequently, various diversity indices and statistical analyses were performed in R (version 4.6.1) using the phyloseq, vegan, and rstatix packages.

Alpha diversity was assessed using Observed Features, Chao1, ACE, Shannon, and Simpson indices. For *E. uniflora*, differences in alpha diversity between young and mature trees and between samples from the two sampling locations were evaluated using Kruskal–Wallis rank-sum tests. The same approach was used to compare alpha diversity among the sampled Myrtaceae species and host genera. Pairwise comparisons were performed using Wilcoxon rank-sum tests, with *p*-values adjusted for multiple comparisons using the Benjamini–Hochberg method. Beta diversity was assessed using Bray–Curtis dissimilarity based on bacterial community abundance data, and Principal Coordinates Analysis (PCoA) was used to visualize differences in community composition. For *E. uniflora*, three complementary PERMANOVA analyses were performed to distinguish the effects of developmental stage and sampling location. First, the effect of developmental stage was tested using all 19 samples with the model Bray–Curtis distance~Age. Second, the effect of sampling location was evaluated among mature trees from Morungava and Porto Alegre using the model Bray–Curtis distance~Location. Third, to assess the effect of developmental stage independently of sampling location, the age comparison was repeated using only the samples collected in Morungava, with the model Bray–Curtis distance~Age. All PERMANOVA analyses were performed using the vegan::adonis2 function with 999 permutations. Analysis of similarities (ANOSIM; vegan::anosim, 999 permutations) was used as a complementary test.

To evaluate whether differences in multivariate dispersion could contribute to the PERMANOVA results, homogeneity of multivariate dispersion was assessed using vegan::betadisper, followed by a permutation test with 999 permutations.

Taxonomic assignments were collapsed to the genus level (sixth taxonomic rank) for downstream analyses including core microbiome identification, Venn diagram visualization, and differential abundance testing with EdgeR (version 4.6.2). Labels in the genus rank that correspond to higher-rank names (family or order names, or clade identifiers inherited from a higher rank) and “uncultured” labels were treated as unclassified at the genus level.

The core microbiome of *Eugenia uniflora* and the Myrtaceae family was defined by plotting the log10 mean relative abundance against occupancy for each bacterial genus in R, following the approach of Shade and Stopnisek (2019) [18]. Genera detected in at least 75% of samples (occupancy ≥ 0.75) were considered part of the core microbiome.

Additionally, Venn diagrams were generated to illustrate shared and unique bacterial genera among the plants, in R using the ggplot2 package. Venn diagrams were constructed considering the presence of bacteria genus in at least one sample per plant group.

Microbial differential abundance analysis was performed using the edgeR package [52] in R to identify bacterial genera associated with developmental stage (old vs. young) in *Eugenia uniflora* and to compare bacterial representation among the Myrtaceae host groups. Raw count data were aggregated at the genus level, filtered to remove low-abundance genera using the filterByExpr function, and normalized using the trimmed mean of M-values (TMM) method. For *E. uniflora*, a negative binomial generalized linear model was fitted with developmental stage as the explanatory factor (~ group), and differential abundance between old and young trees was assessed using a likelihood ratio test (Old vs. Young contrast). Genera with an FDR-adjusted *p*-value < 0.05 were considered differentially represented between developmental stages. For the comparative Myrtaceae analysis, differential abundance was assessed among host groups using mature-tree samples only. Multiple testing was controlled using the Benjamini–Hochberg false discovery rate (FDR) procedure.

### 4.6. Functional Potential Prediction of Bacterial Communities

Functional potential profiles of bacterial communities were predicted using the Tax4Fun [53] R package (version 0.2; https://tax4fun.gobics.de/ (accessed on 13 March 2025))., which infers the metabolic potential of microbial communities based on 16S rRNA gene sequences. The pipeline relies on SILVA-based taxonomic classifications to map taxa to known KEGG Orthologs (KOs), generating a table of predicted gene abundances. The resulting KO abundance table was used to generate box plots of the top functional potential categories across samples using the ggplot2 package in R-4.6.1. These outputs were treated as predictions of functional potential rather than direct measurements of gene abundance, gene expression, or microbial functional activity.

Additionally, ecological functional profiling was also performed using the FAPROTAX database [54] (version 2.0; https://pages.uoregon.edu/slouca/LoucaLab/archive/FAPROTAX2/ (accessed on 25 April 2026)). The ASV table obtained from 16S rRNA sequencing was annotated using the collapse_table.py script, with taxonomy-based mapping to ecological functions. The resulting functional table was used to generate a bar plot of the top functional categories across samples using the ggplot2 package in R-4.6.1.

Differences in metabolic functions between sample groups were assessed using abundance matrices of metabolic pathways or ecological functions. Bray–Curtis distances were calculated using the vegan package in R-4.6.1 to quantify dissimilarities among samples. These distances were visualized using non-metric multidimensional scaling (NMDS, two axes), with confidence ellipses representing the variability within each group. Statistical differences between groups were tested using PERMANOVA (adonis2, vegan) based on the distance matrix. To identify functions that were differentially abundant between groups, log fold change (logFC) and false discovery rate (FDR)-adjusted *p*-values were calculated using edgeR. For visualization of functional patterns, only the most abundant functions (top 10 KOs by total abundance across all samples) were retained.

### 4.7. Microbial Co-Occurrence Network Analysis

Microbiome data were processed and stored using the phyloseq R package [55]. To infer and compare microbial interactions, the NetCoMi R package [56] (version 1.3.0; https://github.com/stefpeschel/NetCoMi (accessed on 1 June 2026)) was employed. Microbiome data were aggregated to the family and genus levels using tax_glom, followed by a rigorous filtering process to remove taxa labeled as “uncultured” or containing non-taxonomic identifiers, ensuring the exclusion of potential noise. Prior to network construction, abundance tables were normalized using the Centered Log-Ratio (CLR) transformation to account for the compositional nature of sequencing data. Microbial association networks for both *Eugenia uniflora* and the mixed Myrtaceae dataset were inferred using the Spearman correlation coefficient, applying a threshold of 0.3 to retain high-confidence associations. The netAnalyze function was utilized to compute key topological metrics, including Degree, Betweenness Centrality, and Closeness, with hub taxa identified based on eigenvector centrality to reflect their relative influence within the community.

## 5. Conclusions

This study demonstrates that host developmental stage is an important driver of phyllosphere bacterial community assembly in *Eugenia uniflora*, influencing bacterial diversity, taxonomic composition, predicted functional potential, and the organization of microbial interaction networks. Mature and young trees harbored distinct bacterial communities, indicating that age-related changes in host physiology contribute not only to microbial recruitment but also to the complexity of microbial interactions. Co-occurrence network analysis further revealed that young plants harbor highly connected and dynamic bacterial communities, whereas mature plants exhibit a more stable microbial core characterized by fewer detectable interactions.

By comparing *E. uniflora* with other *Eugenia* species and three additional Myrtaceae genera, a set of bacterial genera and ecological associations consistently shared among the sampled hosts was identified, suggesting that phyllosphere assembly is structured by both host-specific processes and conserved microbial interactions across the family. The persistence of key co-occurrence patterns across independent networks supports the existence of functionally conserved ecological relationships, despite variation in the bacterial taxa occupying these network positions.

Together, these findings provide new insights into the ecological organization of Myrtaceae phyllosphere microbiomes and establish a foundation for future studies investigating the mechanisms underlying microbial interactions, community stability, and host adaptation. Although the functional significance of these conserved microorganisms and their interactions remain to be experimentally validated, they represent promising targets for research on plant growth promotion, biological control, and microbiome-based strategies for sustainable agriculture. Future studies integrating shotgun metagenomics, metabolomics, microbial isolation, and functional assays will be essential to elucidate the mechanisms governing these plant–microbe interactions and to evaluate their biotechnological potential.

## Figures and Tables

**Table 1:** Core bacterial genera (occupancy ≥ 75%) across the 21 mature Myrtaceae trees, ranked by occupancy and mean relative abundance.

Rank	Bacterial Genera	Occupancy *	Relative Abundance
1	*Hymenobacter*	1.00	0.204
2	*Methylobacterium-Methylorubrum*	1.00	0.179
3	*Sphingomonas*	1.00	0.096
4	1174-901-12	1.00	0.080
5	*Bdellovibrio*	1.00	0.015
6	*Terriglobus*	1.00	0.006
7	*Massilia*	0.95	0.025
8	*Mucilaginibacter*	0.95	0.004
9	*Methylocella*	0.90	0.057
10	*P3OB-42*	0.90	0.016
11	*Acidiphilium*	0.90	0.004
12	*Pseudomonas*	0.86	0.006
13	*Novosphingobium*	0.86	0.005
14	*Spirosoma*	0.86	0.004
15	*Aureimonas*	0.76	0.008
16	*Actinomycetospora*	0.76	0.005
17	*Kineococcus*	0.76	0.002

* Occupancy = proportion of the 21 mature trees in which the genus was detected; 1.00 indicates detection in all trees.

**Table 2:** Sampling information for the Myrtaceae trees analyzed in this study.

Sample ID	Genus	Species	Sampling Location	Coordinates	Tree Age (Years)
Eb_MO3	*Eugenia*	*Eugenia brasiliensis*	Morungava	29°51′52.7″ S 50°53′49.8″ W	15–25
Eb_MO4	*Eugenia*	*Eugenia brasiliensis*	Morungava	29°51′52.9″ S 50°53′49.4″ W	15–25
Ep_MO5	*Eugenia*	*Eugenia pyriformis*	Morungava	29°51′53.5″ S 50°54′51.5″ W	15–25
Es_MO6	*Eugenia*	*Eugenia selloi/nitida*	Morungava	29°51′52.5″ S 50°53′51.5″ W	15–25
Eu_MO1	*Eugenia*	*Eugenia uniflora*	Morungava	29°51′54.1″ S 50°53′48.7″ W	15–25
Eu_MO2	*Eugenia*	*Eugenia uniflora*	Morungava	29°51′52.5″ S 50°53′51.5″ W	15–25
Eu_MY1	*Eugenia*	*Eugenia uniflora*	Morungava	29°51′54.1″ S 50°53′48.7″ W	2
Eu_MY10	*Eugenia*	*Eugenia uniflora*	Morungava	29°51′54.1″ S 50°53′48.7″ W	2
Eu_MY11	*Eugenia*	*Eugenia uniflora*	Morungava	29°51′54.1″ S 50°53′48.7″ W	2
Eu_MY14	*Eugenia*	*Eugenia uniflora*	Morungava	29°51′54.1″ S 50°53′48.7″ W	2
Eu_MY2	*Eugenia*	*Eugenia uniflora*	Morungava	29°51′54.1″ S 50°53′48.7″ W	2
Eu_MY3	*Eugenia*	*Eugenia uniflora*	Morungava	29°51′54.1″ S 50°53′48.7″ W	2
Eu_MY4	*Eugenia*	*Eugenia uniflora*	Morungava	29°51′54.1″ S 50°53′48.7″ W	2
Eu_MY5	*Eugenia*	*Eugenia uniflora*	Morungava	29°51′54.1″ S 50°53′48.7″ W	2
Eu_MY6	*Eugenia*	*Eugenia uniflora*	Morungava	29°51′54.1″ S 50°53′48.7″ W	2
Eu_MY7	*Eugenia*	*Eugenia uniflora*	Morungava	29°51′54.1″ S 50°53′48.7″ W	2
Eu_MY8	*Eugenia*	*Eugenia uniflora*	Morungava	29°51′54.1″ S 50°53′48.7″ W	2
Eu_PO1	*Eugenia*	*Eugenia uniflora*	Porto Alegre	30°4′23″ S 51°7′33″ W	15–25
Eu_PO2	*Eugenia*	*Eugenia uniflora*	Porto Alegre	30°4′23″ S 51°7′33″ W	15–25
Eu_PO3	*Eugenia*	*Eugenia uniflora*	Porto Alegre	30°4′23″ S 51°7′33″ W	15–25
Eu_PO4	*Eugenia*	*Eugenia uniflora*	Porto Alegre	30°4′23″ S 51°7′33″ W	15–25
Eu_PO5	*Eugenia*	*Eugenia uniflora*	Porto Alegre	30°4′23″ S 51°7′33″ W	15–25
Eu_PO6	*Eugenia*	*Eugenia uniflora*	Porto Alegre	30°4′23″ S 51°7′33″ W	15–25
Mp_MO7	*Myrcianthes*	*Myrcianthes pungens*	Morungava	29°51′53.8″ S 50°53′49.6″ W	15–25
Mp_MO8	*Myrcianthes*	*Myrcianthes pungens*	Morungava	29°51′53.8″ S 50°53′49.6″ W	15–25
Ple_MO9	*Plinia*	*Plinia edulis*	Morungava	29°51′53.8″ S 50°53′49.6″ W	15–25
Plt_MO10	*Plinia*	*Plinia trunciflora*	Morungava	29°51′51.7″ S 50°53′53.0″ W	15–25
Plt_MO11	*Plinia*	*Plinia trunciflora*	Morungava	29°51′54.0″ S 50°53′48.3″ W	15–25
Psc_MO12	*Psidium*	*Psidium cattleyanum*	Morungava	29°51′54.1″ S 50°53′48.9″ W	15–25
Psc_MO13	*Psidium*	*Psidium cattleyanum*	Morungava	29°51′51.9″ S 50°53′53.2″ W	15–25
Psg_MO14	*Psidium*	*Psidium guajava*	Morungava	29°51′52.9″ S 50°53′52.0″ W	15–25
Psg_MO15	*Psidium*	*Psidium guajava*	Morungava	29°51′52.6″ S 50°53′50.8″ W	15–25

Abbreviations: M = Morungava; P = Porto Alegre; O = old trees; Y = young trees.

## Data Availability

The raw sequencing data generated in this study have been deposited in the NCBI Sequence Read Archive (SRA) under BioProject accession number PRJNA1122548. All other data supporting the findings of this study are available within the article and its Supplementary Materials.

## Supplementary Materials

The following supporting information can be downloaded at: https://www.mdpi.com/article/10.3390/plants15203138/s1, Figure S1: Taxonomic composition of the *Eugenia uniflora* phyllosphere bacterial community. (A) Relative abundance of major bacterial phyla showing dominance of Proteobacteria, Bacteroidota, and Actinobacteriota. (B) Taxonomic distribution at the family level; Figure S2: Taxonomic composition of the Myrtaceae phyllosphere bacterial community. (A) Relative abundance of major bacterial phyla showing dominance of Proteobacteria, Bacteroidota, and Actinobacteriota. (B) Taxonomic distribution at the family level; Figure S3: Functional profile of the Myrtaceae phyllosphere bacterial community based on FAPROTAX analysis. Bar plots represent the relative abundance of predicted ecological functions across plant samples; Table S1: ASV abundances per sample from *Eugenia uniflora*; Table S2: ASV abundance per sample of Myrtaceae plants; Table S3: Alpha diversity and beta diversity statistical analysis of *Eugenia uniflora* plants; Table S4: Bacterial abundance at the genus level in *Eugenia uniflora* and other Myrtaceae plants; Table S5: Bacterial genera unique or shared across plant ages based on Venn diagram analysis; Table S6: Bacterial genera with differential abundance across *Eugenia uniflora* ages identified by edgeR; Table S7: Metabolic functions across *Eugenia uniflora* plant agespredicted by Tax4Fun; Table S8: Ecological functions across *Eugenia uniflora* plant ages predicted by FAPROTAX; Table S9: Alpha diversity and beta diversity statistical analysis for Myrtaceae plants; Table S10: Bacterial occupancy and relative abundance in the *Eugenia uniflora* microbiome; Table S11: Bacterial occupancy and relative abundance from Myrtaceae microbiome; Table S12: Ecological functions across Myrtaceae predicted by FAPROTAX.
